# Immunogenicity and efficacy of serogroup A and D bacterins against *Pasteurella multocida* in mice

**DOI:** 10.3389/fvets.2023.1132536

**Published:** 2023-03-01

**Authors:** Li-jun Guan, Jin-qian Yang, Qing-yuan Xu, Yi-fan Feng, Xi-chen Zhang, Bo Tang, Zhan-qin Zhao

**Affiliations:** ^1^College of Veterinary Medicine, Jilin University, Changchun, China; ^2^Lab of Veterinary Microbiology, College of Animal Science and Technology, Henan University of Science and Technology, Luoyang, China

**Keywords:** *Pasteurella multocida*, capsular serogroup, immunity, bacterins, cross-protection

## Abstract

**Introduction:**

*Pasteurella multocida* is a widespread respiratory pathogen in pigs, causing swine pneumonia and atrophic rhinitis, and the capsular serogroups A and D are the main epidemic serogroups in infected animals. This study investigated the protective effects of serogroup A and D bacterins against current circulating *P. multocida* strains, to better understand the immunity generated by bacterins.

**Method:**

13 serogroup A (seven A: L3 and six A: L6 strains) and 13 serogroup D (all D: L6 strains) *P. multocida* strains were isolated, and used as inactivated whole cell antigen to prepare *P. multocida* bacterins. Mice were immunized with these bacterins at 21-day interval and intraperitoneally challenged with the homologous and heterologous *P. multocida* strains, respectively. The antibody titer levels and immunization protective efficacy of vaccines were evaluated.

**Results:**

All of the bacterins tested induced high titer levels of immunoglobulin G antibodies against the parental bacterial antigen in mice. Vaccination with the six A: L6 bacterins provided no protection against the parent strain, but some strains did provide heterologous protection against A: L3 strains. Vaccination with the seven A: L3 bacterins provided 50%–100% protection against the parent strain, but none gave heterologous protection against the A:L6 strains. Immunization with the thirteen D: L6 bacterins offered 60%–100% protection against the parent strain, and almost all D: L6 strains gave cross-protection.

**Discussion:**

This study found that the cross-protectivity of serogroup A strains was poor, while serogroup D strains was effective, which provided some insights for *P. multocida* vaccine development.

## Introduction

*Pasteurella multocida*, a facultative anaerobic Gram-negative coccobacillus is named after Louis Pasteur, who first isolated and described this bacterium as the causative agent of fowl cholera in 1880 (1). *P. multocida* infects a wide range of animals and causes pneumonia in pigs, cattle and goats, progressive atrophic rhinitis in swine, fowl cholera, and hemorrhagic septicemia in buffalo and cattle (2). It can spread to humans through cat or dog bites, causing severe zoonotic infections (3–5). The disease type, host specificity, local epidemiology, pathogenicity, and immunogenicity of *P. multocida* are mainly associated with the nature of the virulence factor capsule (6–8). *P. multocida* strains are divided into five serogroups, A, B, D, E, and F based on their capsular antigens (9–11), 16 serotypes (1–16) based on lipopolysaccharide (LPS) antigens (12, 13), and eight LPS genotypes, L1–L8 based on LPS outer core biosynthesis loci (14). These typing schemes have been widely used in epidemiological studies, with combinations of capsular types and LPS genotypes being the most frequently used because of the difficulty of obtaining high-quality antisera (2).

*P. multocida* is responsible for significant economic losses in pig production worldwide (15) and capsular serogroups A and D strains are commonly reported as causing mortality in pigs (16, 17), while capsular types B and F are rarely isolated from pigs (18). The prevalence of the various *P. multocida* serotypes appear to have changed over time in Chinese pigs (19). Prior to the 1990s, swine pasteurellosis was one of the three major infectious diseases (swine fever, swine erysipelas, and swine pasteurellosis) damaging the swine industry in China (20). The dominant types of *P. multocida* were once the capsular serogroups B and A (21, 22), but recent studies suggest that currently the most prevalent serogroups in China are capsular types A and D (23, 24). Inactivated C44-1 aluminum-hydroxide-gel-adjuvanted vaccine, and the derived live EO630 or 679-230 vaccines, have been used for over 50 years to prevent and control swine pasteurellosis in China (25–27). Our previous study showed that the traditional vaccines against swine *P. multocida*, inactivated vaccine (serogroup B strain C44-1) and live vaccine (serogroup B strain EO630), had no protective effect against the epidemic serogroup A and D strains (28), driving a search for new candidate vaccine strains capable of providing immunity against serogroup A and D strains. In this study, 26 inactivated *P. multocida* vaccines were prepared using the currently prevalent serogroup A strains (A1–A13) and serogroup D strains (D1–D13). Mice immunized with these bacterins were challenged with the parent strain and other serogroup A or D strains. Differences in the protective effects of these bacterins showed the cross-protection capacity between serogroup A strains and within serogroup D strains, providing reference results to guide the development of new multivalent *P. multocida* vaccines for pigs.

## Methods

### Bacterial strains and culture conditions

Twenty-six *P. multocida* strains of the capsule serogroups A (strains A1–A13) and D (strains D1–D13) were isolated from diseased swine in central and eastern China by the Veterinary and Biological Products Engineering Laboratory of Henan University of Science and Technology (Luoyang, China), between February 2011 and January 2019 (Table 1) (23). Each strain was cultured on tryptic soy agar (TSA) plates supplemented with 0.1% whole blood with lysed blood cells and 4% healthy bovine serum at 37°C for 12–15 h, to create the F1 generation. The strains were then purified on fresh TSA medium and incubated under aeration at 37°C for up to 12 h to produce the F2 generation use. The isolates were then treated with 15% (v/v) skimmed milk powder and stored at −80°C for future use.

**Table 1:** Virulence results of *P. multocida* serogroup A and D strains in mice.

**Test no**.	**Strain name**	**Date**	**Origin**	**Sample collection**	**Capsular serogroup**	**LPS genotype**	**LD_50_ (CFU)**
1	A1	2013.04	Shandong	Heart blood	A	L3	52
2	A2	2013.04	Henan	Heart blood	A	L3	196
3	A3	2013.01	Henan	Brain	A	L6	238
4	A4	2013.03	Shanxi	Brain	A	L6	74
5	A5	2017.10	Henan	Brain	A	L3	76
6	A6	2017.09	Shanxi	Heart	A	L3	90
7	A7	2013.04	Henan	Heart blood	A	L3	52
8	A8	2012.10	Shanxi	Heart blood	A	L6	32
9	A9	2013.04	Henan	Lung	A	L3	8
10	A10	2013.04	Henan	Lung	A	L3	12
11	A11	2015.12	Shanxi	Lung	A	L6	18
12	A12	2012.11	Henan	Heart blood	A	L6	7
13	A13	2017.09	Shanxi	Brain	A	L6	22
14	D1	2012.06	Henan	Heart blood	D	L6	4.0 × 10^4^
15	D2	2012.07	Shanxi	Lung	D	L6	3.0 × 10^4^
16	D3	2013.03	Shanxi	Lung	D	L6	8.5 × 10^4^
17	D4	2013.04	Henan	Heart blood	D	L6	3.3 × 10^4^
18	D5	2012.02	Henan	Lung	D	L6	1.5 × 10^5^
19	D6	2013.06	Henan	Heart blood	D	L6	5.0 × 10^5^
20	D7	2012.06	Henan	Heart	D	L6	3.6 × 10^5^
21	D8	2012.08	Henan	Lung	D	L6	1.9 × 10^5^
22	D9	2017.09	Shandong	Brain	D	L6	1.8 × 10^5^
23	D10	2019.01	Shanxi	Brain	D	L6	2.3 × 10^5^
24	D11	2012.02	Henan	Lung	D	L6	7.3 × 10^5^
25	D12	2013.06	He nan	Heart blood	D	L6	8.0 × 10^5^
26	D13	2017.09	Jiangsu	Heart blood	D	L6	9.7 × 10^5^

### Animals

Female Kunming (KM) mice aged 5–6 weeks were used for the vaccine study and 10–11 weeks mice were used for the virulence tests. The mice were purchased from the Animal Experiment Center of Zhengzhou University, China, and were housed in groups, provided with food and sterile water *ad libitum*, and acclimated for 3 days before commencing the experiments. The animals in this study were treated in accordance with the recommendations of the Guide for the Animal Care and Use Committee of Henan University of Science and Technology (No. 20220116002).

### Detection of virulence

To determine the virulence of the *P. multocida* strain A1, 25 female KM mice (10–11-weeks-old) were randomly divided into five groups. *P. multocida* strain A1 was grown to a bacterial peak in trypticase soy broth (TSB) with 4% healthy bovine serum and 0.1% whole blood with lysed blood cells, and serially diluted 10-fold in sterile phosphate-buffered saline (PBS) to obtain cultures of ~10–10^4^ CFU/ml. The numbers of bacteria extracted in each dilution were checked using direct-plate viable counts. The first four groups were infected intraperitoneally with 200 μl aliquots of increasing dilutions, and the last group was injected with PBS alone as a control and showed no effects. Groups of mice challenged with the various culture dilutions were isolated and housed in a standard animal facility with *ad libitum* access to a normal rodent diet and water. The inoculated mice were observed for 14 d for signs of disease, and dead mice were dissected immediately to observe their gross pathology. The 50% lethal dose (LD_50_) was calculated on day 14 post-challenge using the Reed-Muench method (29). The other strains were tested in the same way as for strain A1, but the bacterial *P. multocida* serogroup D solutions were serially diluted 10-fold in sterile PBS to obtain cultures of ~10^2^-10^5^ CFU/ml for the challenge tests.

### Inactivated vaccine formulation

The *P. multocida* bacterins of serogroup A and D were prepared in strict accordance with the requirements of the Veterinary Biological Products Regulations of the People's Republic of China, 2000 edition (30). All of the bacterins were prepared in basically the same way as the inactivated A1 aluminum-hydroxide-gel-adjuvanted vaccine (A1 bacterin). A single colony of the F2 generation of the A1 strain was cultured in TSB supplemented with 4% healthy calf serum and 0.1% whole blood with lysed blood cells, and incubated on an oscillator for 15 h at 37°C. The 1% (v/v) mother liquor was then sub-cultured in fresh liquid TSB medium with the same composition for 16 h under the same conditions. The total numbers of bacteria present were calculated from plate counts taken during the growth peak. The whole-cell bacteria were then inactivated with 0.15% formalin for 48 h at 37°C, cultured in TSB overnight, and the resulting inactivated cells were precipitated and washed twice with PBS. The A1 bacterin was produced by emulsifying with aluminum-hydroxide-gel adjuvant. Each dose of bacterin contained 6.0 × 10^9^ dead cells and 167 μg of aluminum-hydroxide-gel in PBS.

### Mice immunization and challenge tests

**Figure 1:**
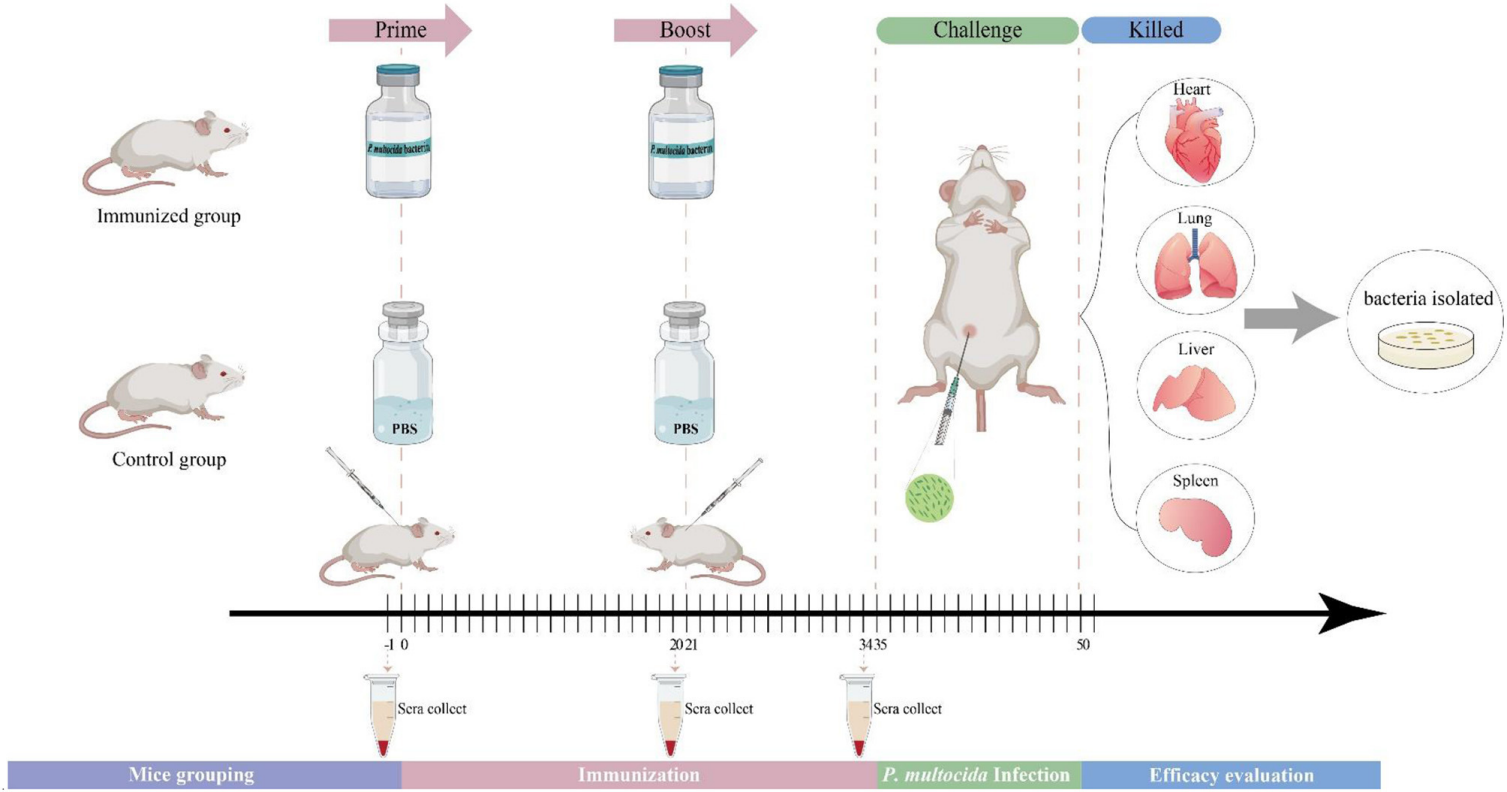
Flow chart of the vaccination, infection, and evaluation procedures of *P. multocida*.

The flow diagram of the immunization procedure is shown in Figure 1. Each group of 7–12 mice were vaccinated subcutaneously with 200 μl of whole-cell vaccine or PBS (control) on day 0 (0.2 ml/mouse), and again on day 21. The mice in the different groups were bred and housed in different cages, isolated from each other. The vaccinated mice were checked for any signs of adverse reactions or disease at 24 h post-vaccination. The vaccinated and control groups were challenged by intraperitoneal injection of 200 μl of the bacterial suspensions containing 4 × the LD_50_ of *P. multocida* virulent strains on day 35. To test the antibody titer levels, blood samples were collected from the tail vein 1 day before primary immunization, booster immunization, and challenge. Survival was observed until 14 days post-challenge, and the daily survival rates after parent and heterologous strain challenges were recorded. The dead mice were necropsied, and substantive organ samples were collected to isolate and identify the bacteria present. The remaining animals were euthanized at the end of the observation period unless otherwise specified herein.

### Serological testing by ELISA

*P. multocida*-specific antibodies were quantified using an enzyme-linked immunosorbent assay (ELISA), as previously described (28). Blood samples were obtained as described above and placed into centrifuge tubes and centrifuged for 2 min at 3,500 rpm. The serum from each mouse was saved in a microcentrifuge tube and stored at −80°C. High-protein-binding 96-well plates (Jet Biofil; TCP011896, Jet Biofil, Guangzhou, China) were coated with 100 μl/well of *P. multocida* serogroup A or D cells suspended in 0.05 M carbonate/bicarbonate buffer (pH 9.6, 100 μl/well) and stored overnight at 4°C. The plates were then washed with ELISA washing buffer (Sangon Biotech, E661005, Shanghai, China) and blocked using 15% (wt/vol) skimmed milk powder in PBS for 1 h at 37°C. The plates were rewashed three times. After the wells had been washed and blocked, the serum antibodies were added to the wells (100 μl/well) and incubated for 30 min at 37°C. The serum samples were then serially diluted two-fold from 1:20 to 1:81,920 in the plates using blocking buffer, and the serially diluted plates were then incubated for 30 min at 37°C. The antibody titers were detected using goat anti-mouse immunoglobulin G (IgG) (Proteintec Group, Inc., Rosemont, IL, USA) diluted in blocking buffer (1:5,000, 100 μl/well), and the sample-treated plates were washed four times and incubated with secondary antibodies for 30 min at 37°C. The plates were washed five times and incubated with TMB chromogenic solution (100 μl/well) for 10 min at room temperature in the dark, and then 100 μl of TMB reaction stop solution was added to each well. Finally, the optical density was measured using an ELISA reader at a wavelength of 450 nm.

### Statistical analysis

Data are expressed as mean ± standard deviation. Differences in the levels of antibodies among all groups of mice were analyzed using one-way analysis of variance (ANOVA). Comparisons of mouse survival rates were analyzed using Fisher's exact test, and survival times were analyzed using the Kaplan-Meier method. All statistical analyses were performed using the SPSS 20.0 software (SPSS Inc., Chicago, IL, USA), and graphics were produced with the Prism 8.0.3 software (Graph Pad, San Diego, CA, USA). Comparisons were considered significantly different if *P* < 0.05 (^*^), *P* < 0.01(^**^), or *P* < 0.001 (^***^).

## Results

### Virulence of the *P. multocida* strains in mice

Descriptions of the various tests are given in full in Table 1. The LD_50_ of serogroup A *P. multocida* strains A1–A13 and serogroup D strains D1–D13 were estimated using the Reed-Muench method (29) in 9–10 weeks old mice (Table 1). The results showed that the LD_50_ values of serogroup A strains were lower than those of serogroup D strains, indicating that the virulence of serogroup A was higher than that of serogroup D strains.

### Antibody responses to vaccination

**Figure 2:**
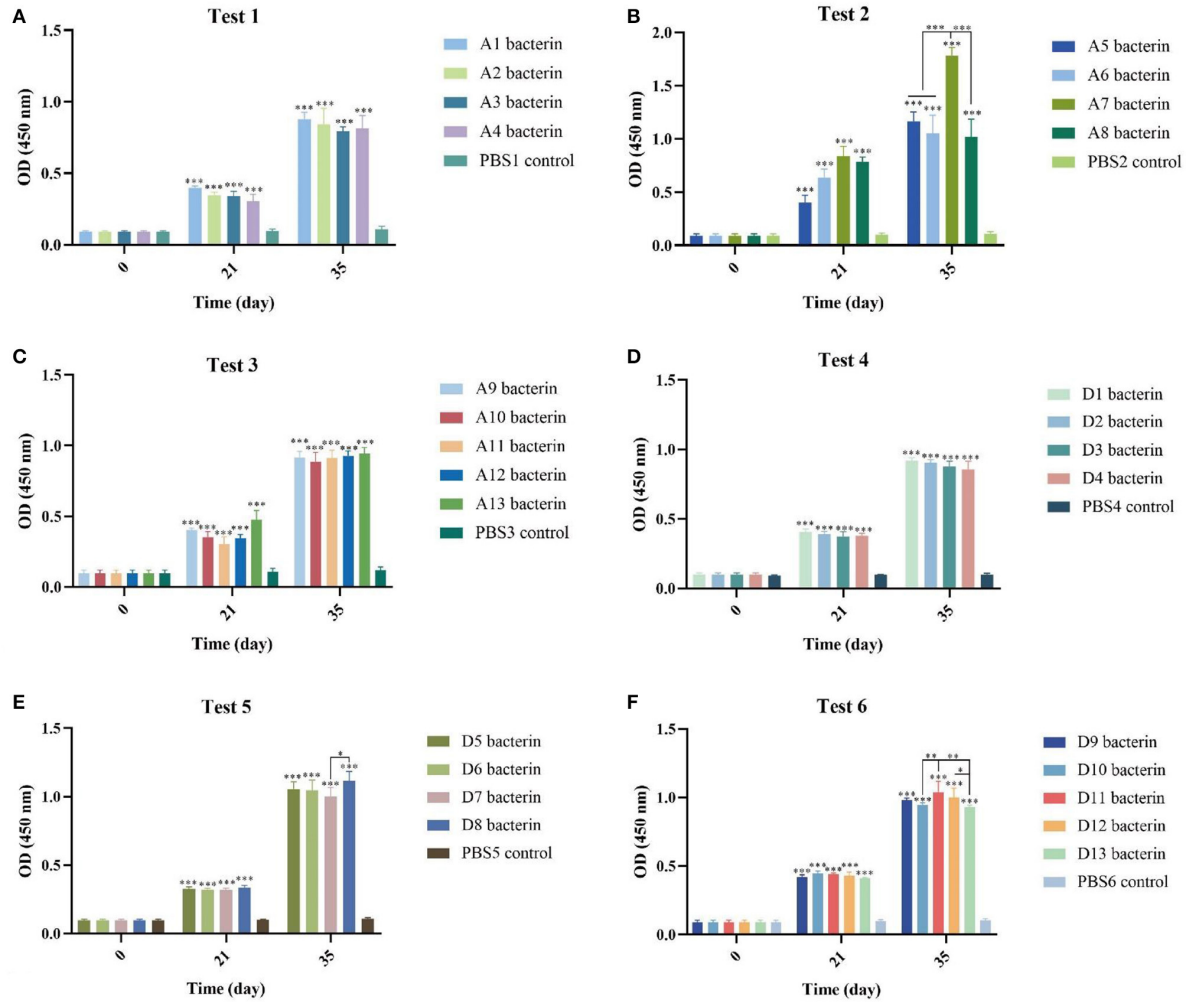
Detection of specific antibodies in mice induced by vaccinations with the different *P. multocida* bacterins. **(A–F)** Antibodies against *P. multocida* in mouse sera at 0-, 21-, and 35-days post vaccination. The error bars represent the SD, with significance levels of *P < 0.05, **P < 0.01, or ***P < 0.001.

We detected the production of IgG antibodies in mouse sera to evaluate the immune response of *P. multocida* serogroups A and D bacterins. At 21-days after the primary vaccination and 14-days after the second immunization, all of the bacterin vaccinated mice produced significantly greater specific serum IgG titers than PBS vaccinated (control) animals (*P* < 0.001) (Figure 2), and after the second immunization all bacterin groups induced the production of higher levels of IgG antibodies compared with the first immunization (*P* < 0.05). In addition, mice revaccinated with A7 bacterin produced a significantly greater serum IgG titer than the other three vaccines in test-2 (*P* < 0.001), the D11 bacterin group being significantly higher than the D10 and D13 bacterin groups after the booster immunization in test-6.

### Immunization protection of A1–A13 bacterins against serogroup A strains

**Figure 3:**
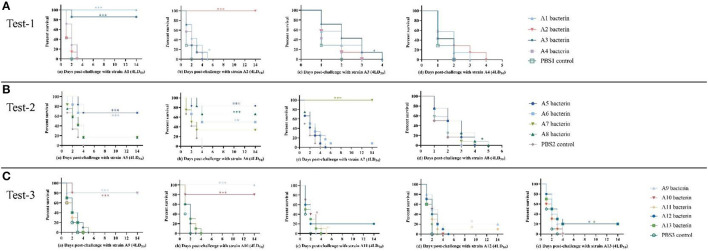
Survival-time curves of mice immunized with A1–A13 bacterins when challenged with serogroup A *P. multocida* strains (Tests-1–3). **(A)** Test-1, mice were subcutaneously immunized with A1–A4 bacterins, and intraperitoneally challenged with 4LD_50_ of strains A1, A2, A3, or A4 (a–d). **(B)** Test-2, mice immunized with A5–A8 bacterins were intraperitoneally challenged with 4LD_50_ of strains A5, A6, A7, or A8 (a–d). **(C)** Test-3, mice immunized with A9–A13 bacterins were intraperitoneally challenged with 4LD_50_ of strains A9, A10, A11, A12, or A13 (a–e). Each group was composed of 7–12 mice and survival time was monitored daily for 14 days after challenge. *P < 0.05; **P < 0.01; ***P < 0.001, in comparison with the PBS control.

The immune protection given by *P. multocida* serogroup A inactivated whole-cell vaccines against type A strains were investigated. KM mice were subcutaneously inoculated with A1–A13 bacterins on day 0, and day 21. On day-14 after the booster immunization, mice were intraperitoneally challenged with serogroup A strains of *P. multocida*. The test-1 result showed that vaccination with A1 or A2 bacterin provided the mice with 100% protection against challenge with 4 × the LD_50_ of the parent strain, and that vaccination with A3 bacterin provided 86% protection against a 4LD_50_ challenge with strain A1 (Table 2). Challenge of A1 or A3 bacterin vaccinated animals with A1 showed improved survival times compared with the control vaccinated animals (*P* < 0.001), and mice vaccinated with A2 bacterin showed significant differences in survival time compared with the A2 challenged controls (Figure 3A). However, mice immunized with A1, A2, A3, or A4 bacterin received no protection against a lethal challenge with strains A3 and A4, and A1 bacterin could not protect mice against strain A2 (Table 2). All of the animals vaccinated with the A2 bacterin and infected with strains A1, A3, or A4 died within 5 days post-infection (Figure 3A), the protection rate being zero.

**Table 2:** Protection rate of A1–A4 bacterins against *P. multocida* strains (Test-1).

**Immunization Groups**	**Antigen concentration (CFU/mL)**	**Immune dose (mL)**	**Challenge strains (A:L3/L6) and survival (challenge bacteria content)**					
			**A1 (A:L3)**	**A2 (A:L3)**	**A3 (A:L6)**	**A4 (A:L6)**	**Total survival**	**Total survival rate**
			**(4LD**_50_ **)**	**(4LD**_50_ **)**	**(4LD**_50_ **)**	**(4LD**_50_ **)**		
A1 bacterin	6.0 × 10^9^	0.2	7/7^a^	0/7^b^	0/7^b^	0/7^b^	7/28^a^	25%
A2 bacterin	6.0 × 10^9^	0.2	0/7^b^	7/7^a^	0/7^b^	0/7^b^	7/28^a^	25%
A3 bacterin	6.0 × 10^9^	0.2	6/7^a^	0/7^b^	0/7^b^	0/7^b^	6/28^a^	21%
A4 bacterin	6.0 × 10^9^	0.2	0/7^b^	0/7^b^	0/7^b^	0/7^b^	0/28^b^	0
PBS1 control	–	0.2	0/7^b^	0/7^b^	0/7^b^	0/7^b^	0/28^b^	0

The shaded value refers to the protection rate of A5, A6, A7, and A8 bacterins against the parent strain. Comparison of the difference of protective effect of different immune groups at the same challenge dose, the same letter (a or b) in the upper right corner of the value indicates that there is no difference in the protection rate between groups (*P* > 0.05), and different letters (a and b) indicate significant difference (*P* < 0.05).

The test-2 result indicated that vaccination with A5, A6, or A7 bacterin gave protection against the parent strain, with survival rates of 67%, 50%, and 100%, respectively, while mice immunized with A8 bacterin did not survive challenge with the parent strain (Table 3). Mice immunized with A5 bacterin and challenged with A6 had an 83% survival rate, and those immunized with A6 bacterin received 67% protection against an A5 challenge (Table 3). Meanwhile, the survival time of mice in the two immune groups was longer compared with the corresponding control group (*P* < 0.001) (Figure 3B), indicating a cross-protective immunity between the two strains. Interestingly, 67% of mice vaccinated with A8 bacterin went on to survive when challenged with A6, but the A6 bacterin could not protect them against challenge with A8, with no statistically difference between the A6 bacterin challenge and the corresponding control group (*P* > 0.05) (Figure 3B). In addition, the immune protection rates and survival times after challenge showed no cross-protection among strains A5, A7, and A8.

**Table 3:** Protection rate of A5–A8 bacterins against *P. multocida* strains (Test-2).

**Immunization Groups**	**Antigen concentration (CFU/mL)**	**Immune dose (mL)**	**Challenge strains (A:L3/L6) and survival (challenge bacteria content)**					
			**A5 (A:L3)**	**A6 (A:L3)**	**A7 (A, L3)**	**A8 (A:L6)**	**Total survival**	**Total survival rate**
			**(4LD**_50_ **)**	**(4LD**_50_ **)**	**(4LD**_50_ **)**	**(4LD**_50_ **)**		
A5 bacterin	6.0 × 10^9^	0.2	8/12^a^	10/12^a^	0/12^b^	0/12^b^	18/48^a^	38%
A6 bacterin	6.0 × 10^9^	0.2	8/12^a^	6/12^a^	1/12^b^	0/12^b^	15/48^a^	31%
A7 bacterin	6.0 × 10^9^	0.2	2/12^b^	4/12^b^	12/12^a^	0/12^b^	18/48^a^	38%
A8 bacterin	6.0 × 10^9^	0.2	2/12^b^	8/12^a^	0/12^b^	0/12^b^	10/48^a^	21%
PBS2 control	–	0.2	0/12^b^	0/12^b^	0/12^b^	0/12^b^	0/48^b^	0

The shaded value refers to the protection rate of A5, A6, A7, and A8 bacterins against the parent strain. Comparison of the difference of protective effect of different immune groups at the same challenge dose, the same letter (a or b) in the upper right corner of the value indicates that there is no difference in the protection rate between groups (*P* > 0.05), and different letters (a and b) indicate significant difference (*P* < 0.05).

The test-3 result showed that only the A9 and A10 bacterins could protect against infection with the parent strain, both providing survival rates of 80%. Vaccination with A9 bacterin could provide 100% protection against a challenge with strain A10, and 80% of the mice vaccinated with A10 bacterin survived when challenged with strain A9. Moreover, the survival time of mice in both immunized groups was clearly and significantly prolonged (*P* < 0.001) relative to the control group, all of which died within 4 days (Figure 3B), indicating cross-protection between strains A9 and A10 (Table 4). In addition, there was little cross-protection among strains A11, A12, and A13 (Table 3, Figure 3C).

**Table 4:** Protection rate of A9–A13 bacterins against *P. multocida* strains (Test-3).

**Immunization Groups**	**Antigen concentration (CFU/ml)**	**Immune dose (ml)**	**Challenge strains (A:L3/L6) and survival (challenge bacteria content)**					
			**A9 (A:L3)**	**A10 (A:L3)**	**A11 (A:L6)**	**A12 (A:L6)**	**A13 (A:L6)**	**Total survival**	**Total survival rate**
			**(4LD**_50_ **)**	**(4LD**_50_ **)**	**(4LD**_50_ **)**	**(4LD**_50_ **)**	**(4LD**_50_ **)**		
A9 bacterin	6.0 × 10^9^	0.2	8/10^a^	10/10^a^	0/10^b^	2/10^b^	0/10^b^	20/50^a^	40%
A10 bacterin	6.0 × 10^9^	0.2	8/10^a^	8/10^a^	0/10^b^	0/10^b^	0/10^b^	16/50^a^	32%
A11 bacterin	6.0 × 10^9^	0.2	0/10^b^	0/10^b^	0/10^b^	1/10^b^	0/10^b^	1/50^b^	2%
A12 bacterin	6.0 × 10^9^	0.2	0/10^b^	0/10^b^	2/10^b^	0/10^b^	2/10^b^	4/50^b^	8%
A13 bacterin	6.0 × 10^9^	0.2	0/10^b^	0/10^b^	0/10^b^	0/10^b^	2/10^b^	2/50^b^	4%
PBS3 control	–	0.2	0/10^b^	0/10^b^	0/10^b^	0/10^b^	0/10^b^	0/50^b^	0

The shaded value refers to the protection rate of A9, A10, A11, A12 and A13 bacterins against the parent strain. Comparison of the difference of protective effect of different immune groups at the same challenge dose, the same letter (a or b) in the upper right corner of the value indicates that there is no difference in the protection rate between groups (*P* > 0.05), and different letters (a and b) indicate significant difference (*P* < 0.05).

### Immunization with serogroup D bacterins can protect mice challenged with serogroup D strains

**Figure 4:**
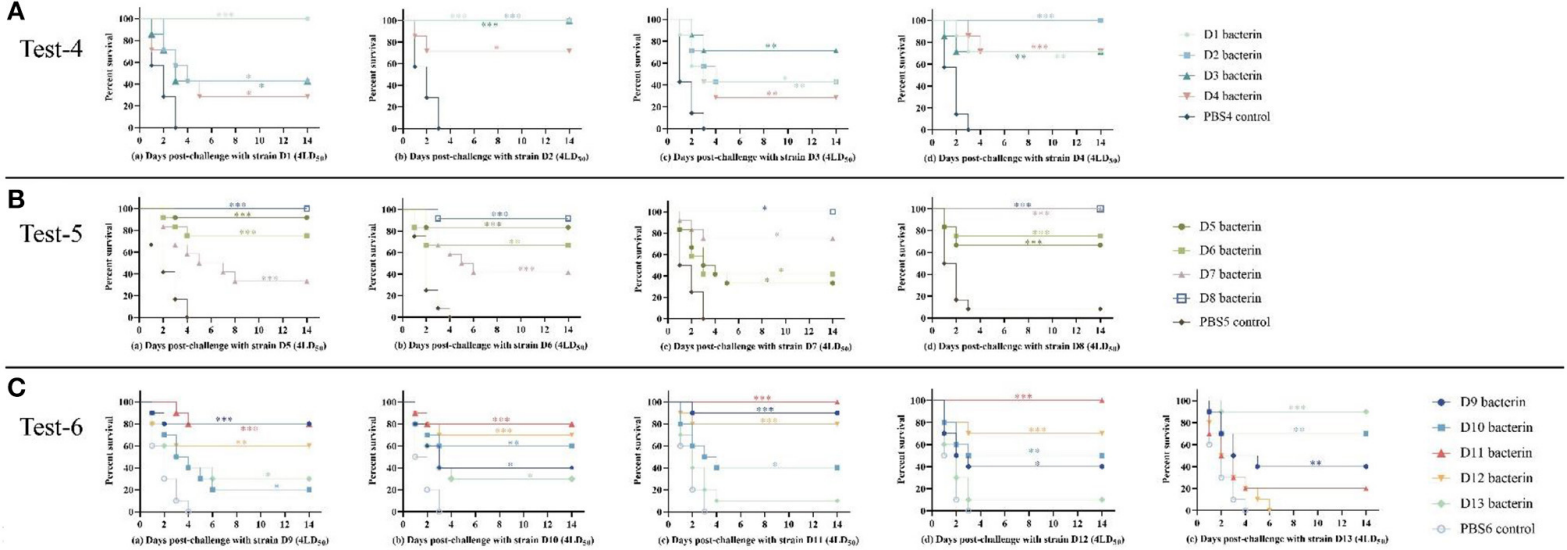
Survival-time curves of mice immunized with the D1–D13 bacterins when challenged with serogroup D *P. multocida* strains (Test-1–3). **(A)** Test-4, mice were subcutaneously immunized with the D1–D4 bacterins, and intraperitoneally challenged with (a–d) 4LD_50_ of strain D1, D2, D3, or D4. **(B)** Test-5, mice immunized with the D5–D8 bacterins were intraperitoneally challenged with (a–d) 4LD_50_ of strain D5, D6, D7, or D8. **(C)** Test-6, mice immunized with the D9–D13 bacterins were intraperitoneally challenged with (a–e) 4LD_50_ of strain D9, D10, D11, D12, or D13. Each group was composed of 7–12 mice and survival time was monitored daily for 14 days after challenge. *P < 0.05; **P < 0.01; ***P < 0.001, compared with the PBS control.

To determine the protection against parent and heterologous *P. multocida* strain infections given by inoculation with serogroup D *P. multocida* bacterins, mice were vaccinated with D1–D13 inactivated whole-cell vaccine at 21-day intervals and intraperitoneally challenged with different serogroup D strains 14 days after the booster vaccination. In test-4, vaccination with D1, D2, D3, or D4 bacterin protected mice against 4LD_50_ challenge with the parent strain, providing protection rates of 100%, 100%, 71%, and 71%, respectively (Table 5). The results indicated that D1, D2, D3, or D4 bacterin provided protection against infection by the parent strain. Cross-protection was observed among strains D1, D2, D3, and D4; the protective efficiency of D1 and D3 bacterins against the D2 strain was 100%, and that of D2 bacterin against the D4 strain was 100% (Table 5). The percentage survival in test-4 is shown in Figure 4. All control group mice succumbed to disease by 3 days post-infection. Even though there were no statistically significant differences in the protection rates of D2, D3, and D4 bacterins against strain D1, the survival time of vaccinated mice was significantly longer than that of the control group (*P* < 0.05). Similarly, mice immunized with D1, D2, and D4 bacterins also showed significantly increased survival times (*P* < 0.05) when challenged with strain D3 (Figure 4A).

**Table 5:** Protection rate of D1–D4 bacterins against *P. multocida* strains (Test-4).

**Immunization groups**	**Antigen concentration (CFU/ml)**	**Immune dose (ml)**	**Challenge strains (A:L3/L6) and survival (challenge bacteria content)**					
			**D1 (D:L6)**	**D2 (D:L6)**	**D3 (D:L6)**	**D4 (D:L6)**	**Total survival**	**Total survival rate**
			**(4LD_50_)**	**(4LD_50_)**	**(4LD_50_)**	**(4LD_50_)**		
D1 bacterin	6.0 × 10^9^	0.2	7/7^a^	7/7^a^	3/7^ab^	5/7^a^	22/28^a^	79%
D2 bacterin	6.0 × 10^9^	0.2	3/7^ab^	7/7^a^	3/7^ab^	7/7^a^	20/28^a^	71%
D3 bacterin	6.0 × 10^9^	0.2	3/7^ab^	7/7^a^	5/7^a^	5/7^a^	20/28^a^	71%
D4 bacterin	6.0 × 10^9^	0.2	2/7^b^	5/7^a^	2/7^ab^	5/7^a^	14/28^a^	50%
PBS4 control	–	0.2	0/7^b^	0/7^b^	0/7^b^	0/7^b^	0/28^b^	0

The shaded value refers to the protection rate of D1, D2, D3, and D4 bacterins against the parent strain. Comparison of the difference of protective effect of different immune groups at the same challenge dose, the same letter (a or b) in the upper right corner of the value indicates that there is no difference in the protection rate between groups (*P* > 0.05), and different letters (a and b) indicate significant difference (*P* < 0.05).

In test-5, mice immunized with D5, D6, D7, or D8 bacterin were challenged with 4LD_50_ of the parent strain, with survival rates of 92%, 67%, 75%, and 100%, respectively (Table 6). The results showed that the D5, D6, D7, and D8 bacterins provided protection against the parent strain. In addition, there was cross-protection among strains D5, D6, D7, and D8; the D7 bacterin provided 100% protective efficacy against challenge with strain D8, and the protective efficacy of D8 bacterin against strain D5 was 100% (Table 6). No significant protection was conferred by the D7 bacterin against exposure to challenge strain D5 (*P* > 0.05), however, mice immunized with D7 showed longer survival times (*P* < 0.05) (Figure 4B). The D5 bacterin showed the same protection result after challenge with strain D7, and there were significant differences in survival time between the D5 bacterin vaccinated group and the control group (*P* < 0.05) (Figure 4B).

**Table 6:** Protection rate of D5–D8 bacterins against *P. multocida* strains (Test-5).

**Immunization groups**	**Antigen concentration (CFU/ml)**	**Immune dose (ml)**	**Challenge strains (A:L3/L6) and survival (challenge bacteria content)**					
			**D5 (D:L6)**	**D6 (D:L6)**	**D7 (D:L6)**	**D8 (D:L6)**	**Total survival**	**Total survival rate**
			**(4LD**_50_ **)**	**(4LD**_50_ **)**	**(4LD**_50_ **)**	**(4LD**_50_ **)**		
D5 bacterin	6.0 × 10^9^	0.2	11/12^a^	10/12^Aa^	4/12^ab^	8/12^a^	33/48^Aa^	69%
D6 bacterin	6.0 × 10^9^	0.2	9/12^Aa^	8/12^Aa^	5/12^a^	9/12^a^	31/48^Aa^	65%
D7 bacterin	6.0 × 10^9^	0.2	4/12^Ab^	5/12^A^	9/12^a^	12/12^a^	30/48^A^	63%
D8 bacterin	6.0 × 10^9^	0.2	12/12^a^	11/12^a^	6/12^a^	12/12^a^	41/48^a^	85%
PBS5 control	–	0.2	0/12^b^	0/12^b^	0/12^b^	1/12^b^	1/48^b^	2%

The shaded value refers to the protection rate of D5, D6, D7, and D8 bacterins against the parent strain. Comparison of the difference of protective effect of different immune groups at the same challenge dose, the same letter (a, b, or A) in the upper right corner of the value indicates that there is no difference in the protection rate between groups (*P* > 0.05), and different letters (a, b, and A) indicate significant difference (*P* < 0.05).

In test-6, vaccination with D9, D10, D11, D12, or D13 bacterin provided 80%, 60%, 100%, 70%, and 90% protection against lethal challenge with the parent strain, respectively (Table 7). As shown in Figure 4, the mice in the control groups died within 4 days of *P. multocida* infection. The protective efficiency of bacterins against challenge strains and the survival curves showed a cross-protection effect between strains. Vaccination with the D11 bacterin resulted in 20% survival when challenged with strain D13, and the D13 bacterin resulted in 10% survival when challenged with strain D11 (Table 7), with no differences in survival time between the vaccinated and control groups (*P* > 0.05) (Figure 4C), indicating no cross-protection between the D11 and D13 strains. Mice vaccinated with the D12 bacterin were not protected against strain D13, and the protection rate of the D13 bacterin against strain D12 was only 10% (Table 7), with no differences in survival time between the vaccinated and control groups (*P* > 0.05) (Figure 4C), showing that there was no cross-protection between the D12 and D13 strains.

**Table 7:** Protection rate of D9–D13 bacterins against *P. multocida* strains (Test-6).

**Immunization groups**	**Antigen concentration (CFU/ml)**	**Immune dose (ml)**	**Challenge strains (D:L6) and survival (challenge bacteria content)**					
			**D9 (D:L6)**	**D10 (D:L6)**	**D11 (D:L6)**	**D12 (D:L6)**	**D13 (D:L6)**	**Total survival**	**Total survival rate**
			**(4LD**_50_ **)**	**(4LD**_50_ **)**	**(4LD**_50_ **)**	**(4LD**_50_ **)**	**(4LD**_50_ **)**		
D9 bacterin	6.0 × 10^9^	0.2	8/10^Aa^	4/10^Ab^	9/10^Aa^	4/10^Bb^	4/10^ab^	29/50^aB^	58%
D10 bacterin	6.0 × 10^9^	0.2	2/10^Bb^	6/10^Aa^	4/10^Ab^	5/10^AB^	7/10^Aa^	24/50^ABc^	48%
D11 bacterin	6.0 × 10^9^	0.2	8/10^Aa^	8/10^a^	10/10^a^	10/10^a^	2/10^Ab^	38/50^a^	76%
D12 bacterin	6.0 × 10^9^	0.2	6/10^AaB^	7/10^Aa^	8/10^Aa^	7/10^aB^	0/10^b^	28/50^ac^	56%
D13 bacterin	6.0 × 10^9^	0.2	3/10^Ab^	3/10^Ab^	1/10^b^	1/10^Ab^	9/10^a^	17/50^A^	34%
PBS6 control	–	–	0/10^b^	0/10^b^	0/10^b^	0/10^b^	0/10^b^	0/50^b^	0

The shaded value refers to the protection rate of D9, D10, D11, D12, and D13 bacterins against the parent strain. Comparison of the difference of protective effect of different immune groups at the same challenge dose, the same letter (a, b, A, or B) in the upper right corner of the value indicates that there is no difference in the protection rate between groups (*P* > 0.05), and different letters (a, b, A, and B) indicate significant difference (*P* < 0.05).

## Discussion

*P. multocida* is a recognized primary pathogen causing economically significant diseases, with a serious impact on the health and economics of the pig-rearing industry worldwide (31). The control of *P. multocida* infections using antimicrobial agents is often difficult due to the prevalence of antibiotic-resistant strains and the adaptability of this pathogen (32). Prophylactic vaccination provides one possible approach to early eradication of *P. multocida* outbreaks (3). In Europe and China, all of the licensed *P. multocida* vaccines are bacterins (33). *P. multocida* capsular serogroup A is the most common serogroup found in every host category, including poultry, cattle, sheep, pigs, humans, and cats (34–39). The most frequent *P. multocida* strains associated with swine pasteurellosis in China are the A and D capsular types (19, 23, 24), and L6 and L3 LPS genotype (24, 40). However, in China the most widely used and marketed *P. multocida* bacterin is whole-cell formalin-killed *P. multocida* C44-1 aluminum-hydroxide-gel-adjuvanted vaccine, a capsular serogroup B antigen, which in our previous study failed to protect mice against the serogroup A and D strains in current circulation (28). This study therefore investigated the protective efficacy of bacterins prepared from the currently epidemic serogroup A and D strains to find candidate vaccine strains.

In this report, we determined the LD_50_ of 26 *P. multocida* strains, and found that the virulence of *P. multocida* serogroup A strains was greater than D strains. The reason for the difference in virulence may be related to divergent expression of the virulence gene of *P. multocida*, which might contribute to adhesion, colonization, and invasion (41, 42). To explore the immune responses of *P. multocida* serogroups A and D bacterins, serum was collected before each immunization. The immune response associated with *P. multocida* infection is predominantly humoral rather than cell-mediated (43, 44), and inactivated vaccines can induce strong humoral responses (45). Further, aluminum adjuvant is a potent immunomodulator and a strong Th2 stimulant, advantageous properties in a good vaccine against extracellular pathogens such as *P. multocida* (46). Hence, all of the serum samples collected in the present research were tested using IgG-based ELISA. The results showed that the vaccine groups tested generated high levels of IgG titer, and that antibody titers were highest after secondary immunization. Moreover, there were statistical differences between the antibody levels produced by some of bacterins, suggesting an interaction between the immune response of an individual animal and the selected vaccine strain.

Mice were intraperitoneally challenged with virulent *P. multocida* strains after a second bacterin vaccination. Seven of the 13 A bacterins tested produced protective effects against the parent strains. Intriguingly, the antigen strains present in the seven effective serogroup A bacterins all shared the L3 genotype. Moreover, the protective immunity conferred by serogroup A bacterins against heterologous strains showed that cross-protection occurred between strains A5 and A6, and between strains A9 and A10, all four of which were also genotype L3. These results begged the question, why does the A:L6 genotype strain not give immunological protection against the parent strain and other serogroup A strains? These findings indicate that the protective immunity conferred by vaccination with inactivated *P. multocida* strains is dependent on their LPS structure. LPS is one of the primary virulence factors of *P. multocida* and displays significant structural variability across different *P. multocida* strains (47). Harper et al. have shown that inactivated whole-cell vaccines give protection only against strains with identical, or highly similar, LPS structures (48).

Additionally, A3 bacterin (A:L6) provided 86% protection against a challenge with strain A1 (A:L3), while A1 bacterin gave no protection against strain A3. A8 bacterin (A:L6) gave 67% or 17% protection against strains L3, A6, and A5, respectively, while A6 or A5 gave no protection against A8. These results indicated that even though the L6 type bacterin used in this study gave protection against the L3 type strain, the L3 type bacterin in turn was unable to protect mice against challenge with the L6 type strain. This may be related to the presence of multiple LPS structures generated by spontaneous mutations in the LPS biosynthesis genes in L3 or L6 type strains (49), and that the structural diversity of L6 type strains may be more complex than that of L3 strains (49, 50). This finding suggested that while L6 type bacterin protects mice against the type L3 strain, L3 type bacterin may not protect mice against the L6 strain. However, while the L6 type bacterin gave no immune protection against the L3 strain, L3 type bacterin gave no immune protection against the L6 strain. This assumption was confirmed regarding the genotype A:L6 strains and genotype A:L3 strains in this study, e.g., between strains A8 and A7, between strains A12 and A10, between strains A13 and A9 (A10), and between strains A11 and A10.

The serogroup D inactivated vaccine tests showed that all bacterins provided immune protection against the parental strain, and in 57 challenge tests between the serogroup D bacterin groups and the serogroup D challenge strains, no statistical differences in survival rates were observed in 19 groups compared with the control group (Tables 5–7). However, combined with the analysis of the survival-time curves of immunized mice after challenge, only four groups showed no difference in survival time compared with the control. These findings showed that almost all serogroup D bacterins used in this study offered heterologous protection, but no cross-protective effects were shown between strains D13 and D11, or between strains D13 and D12. In addition, even though the survival times of the remaining 15 immunized groups were prolonged, the protection rate was weak, which may be related to the structural diversity of the LPS outer cores, or the levels of virulence gene expression in *in vitro* and *in vivo* situations (41).

In summary, this study generated 26 *P. multocida* bacterins from the currently prevalent serogroup A and D strains and assessed their potential efficacy as inactivated vaccines. None of the six *P. multocida* genotype A:L6 strains could provide effective homologous protection, but two of them could provide heterologous protection against the genotype A:L3 challenge strains. All seven of the genotype A:L3 strains produced some degree of homologous protection, but none provided heterologous protection against genotype A:L6 strains. Almost all of the *P. multocida* genotype D:L6 strains provided homologous protection and heterologous protection. These findings provided insights into the effectiveness of bacterins as vaccinations against *P. multocida*, and provided some baseline references for the development of efficacious bivalent vaccines.

## Data availability statement

The original contributions presented in the study are included in the article/supplementary material, further inquiries can be directed to the corresponding authors.

## Ethics statement

All procedures performed in studies involving animals were approved by the Animal Care and Use Committee of Henan University of Science and Technology (No. 20220116002).

## Author contributions

L-jG and Z-qZ conceived and designed the study. L-jG and J-qY performed the experiments. L-jG, Q-yX, and Y-fF analyzed the data. L-jG, X-cZ, BT, and Z-qZ wrote and revised the manuscript. All authors read and approved the final version.
